# Assessment of Pain During Nerve Conduction Studies in Patients With Carpal Tunnel Syndrome

**DOI:** 10.1016/j.jhsg.2021.12.004

**Published:** 2022-01-05

**Authors:** Toru Sasaki, Akimoto Nimura, Tomoyuki Kuroiwa, Takafumi Koyama, Atsushi Okawa, Koji Fujita

**Affiliations:** ∗ Department of Orthopaedic and Spinal Surgery, Graduate School of Medical and Dental Sciences, Tokyo Medical and Dental University, Tokyo, Japan; † Department of Functional Joint Anatomy, Graduate School of Medical and Dental Sciences, Tokyo Medical and Dental University, Tokyo, Japan

**Keywords:** Carpal tunnel syndrome, Nerve conduction study, Pain, Visual analog scale score

## Abstract

**Purpose:**

Patients with suspected carpal tunnel syndrome (CTS) often undergo nerve conduction studies (NCSs). Although patients sometimes complain of NCS-related discomfort, including severe pain, pain evaluations during such NCSs are lacking. We aimed to measure the pain experienced by patients with CTS during NCSs.

**Methods:**

This prospective study included 30 patients with CTS who underwent NCSs between April 2018 and March 2019. Pain because of electrical stimulation during NCSs was evaluated using a visual analog scale, and we statistically analyzed pain-related factors such as age, sex, complications, severity grading scale, the intensity of maximum stimulation, and examination time.

**Results:**

The mean visual analog scale score for NCSs was 5.2, and the visual analog scale score increased as the intensity of maximum stimulation and examination time increased.

**Conclusions:**

We measured the pain because of electrical stimulation experienced by patients with CTS during NCSs. Our findings indicate that medical staff must be mindful of the potential pain experienced by patients during NCSs and educate patients regarding the necessity of the examination and its procedures.

**Type of study/level of evidence:**

Diagnostic Ⅳ.

In carpal tunnel syndrome (CTS), the most common nerve entrapment syndrome, the median nerve is entrapped at the wrist. Patients with CTS often undergo nerve conduction studies (NCSs). Nerve conduction studies can objectively evaluate nerve function and severity of neuropathy and help determine the indication for surgery and assess postoperative function.^1^

However, some reports have suggested that NCSs are unnecessary for CTS because CTS can be diagnosed solely based on physical examinations and clinical symptoms.^2^^,^^3^ Furthermore, patients may complain of discomfort, such as severe pain, after NCSs. Various reports have been published on the discomfort and anxiety experienced by patients who underwent EMG and NCSs.^4^, ^5^, ^6^, ^7^, ^8^ However, most of these prior studies did not consider the disease conditions of patients and the examination details. When performing NCS, we should inform the patients of the details of the test, but there are insufficient data on discomfort, such as pain, during NCSs.

In this study, we hypothesized that the intensity of electrical stimulation, examination time, and patient background would affect pain caused by NCS. In this study, we aimed to measure the pain experienced by patients with CTS during NCSs and statistically analyze factors related to the pain that should be recognized by the medical staff.

## Materials and Methods

### Patients

This study was approved by our institutional review board, and we obtained written informed consent from all patients. All procedures in this study were performed in accordance with the Declaration of Helsinki. This prospective study included 30 patients suspected by hand surgeons of having CTS and who underwent NCSs between April 2018 and March 2019. All subjects met the following inclusion criteria: (1) symptoms of CTS (numbness, tingling, and pain); (2) CTS confirmed on physical examination by a positive Phalen or Tinel sign; and (3) CTS confirmed by NCS (prolonged distal motor latencies of the median nerve of >4.5 ms or sensory nerve conduction velocity of <40 m/s).^9^ The patients who had previously undergone a carpal tunnel release surgery or those with comorbidities, such as diabetes mellitus, were also included. In addition to patients who underwent testing with the conventional NCS method, we included patients who underwent NCS testing with other methods, such as the second lumbrical-interosseous comparison method.^10^ The following exclusion criteria were applied: (1) patients who had undergone NCSs in only 1 hand; (2) patients who could not answer the questionnaire immediately after the examination; (3) patients aged <20 years; and (4) non-Japanese speakers.

### Nerve conduction studies

Nerve conduction studies were performed on both hands by trained clinical technicians, with the patients in the supine position and a relaxed state. The skin temperature of 32 °C was obtained on the dorsum of the hand. Nerve conduction study recordings of the bilateral median nerve were performed using an evoked potential/EMG system (MEB-2300; Nihon Kohden) with a bandpass filter set at 10 Hz to 5 kHz for motor nerve recording and at 20 Hz to 2 kHz for sensory recording. In sensory NCSs, the sensory nerve action potential was antidromically recorded with a pair of cup electrodes placed over the distal and proximal interphalangeal joints of the index finger. Square-pulse supramaximal electrical stimulation was delivered at the palm, wrist, and elbow. We used palm stimulation to diagnose local conduction disorders in our hospital and calculated the sensory nerve velocity between the palm and wrist. In motor NCSs, the compound muscle action potential was recorded with a pair of surface cup electrodes placed over the abductor pollicis brevis muscle. Square-pulse supramaximal electrical stimulation was delivered at the wrist and elbow. Sensory and motor nerve action potentials were measured separately. Each stimulation was performed twice to confirm reproducibility (frequency, 0.5 Hz; duration, 0.3 ms). We set the maximum stimulation as the intensity at which the amplitude of the waveform does not change even when the stimulus intensity is increased. For severe cases in which the recordings were not derived using these conventional methods, a second lumbrical-interosseous comparison method was employed, and such cases were also included in this study. The NCS results were classified according to the Bland classification, which is based on electrophysiological severity.^9^

### Statistical analysis

Pain because of electrical stimulation during the NCSs was evaluated using the visual analog scale (VAS) immediately after the examination.

Based on previous study, we considered a score of 2.0 on the VAS for pain as clinically meaningful when identifying differences in NCS pain.^8^ With a sample of 30 participants (15 participants per group), we calculated that the study would have 80% power to detect a score of 2.0 on the VAS for pain, with a type 1 error of 5%. We used a standard deviation of a 2.1 score on the VAS for pain for the power analysis based on data reported by the previous study.^8^

We analyzed factors related to NCS-induced pain. The Mann-Whitney U test was used to identify differences between 2 independent groups for the following factors: age (<65 years, ≥65 years), sex (male, female), diabetes mellitus, the severity of CTS based on the grading scale (Bland classification grades 0–3, grades 4–6), the intensity of maximum stimulation (<40 mA, ≥40 mA), and examination time (<30 min, ≥30 min).^11^^,^^12^ Examination time was defined as the duration from the beginning to the end of the last stimulation. *P* < .05 was considered to reflect statistical significance. We added multiple regression analysis for items that showed a significant difference in the Mann-Whitney U test.

## Results

**Figure 1:**
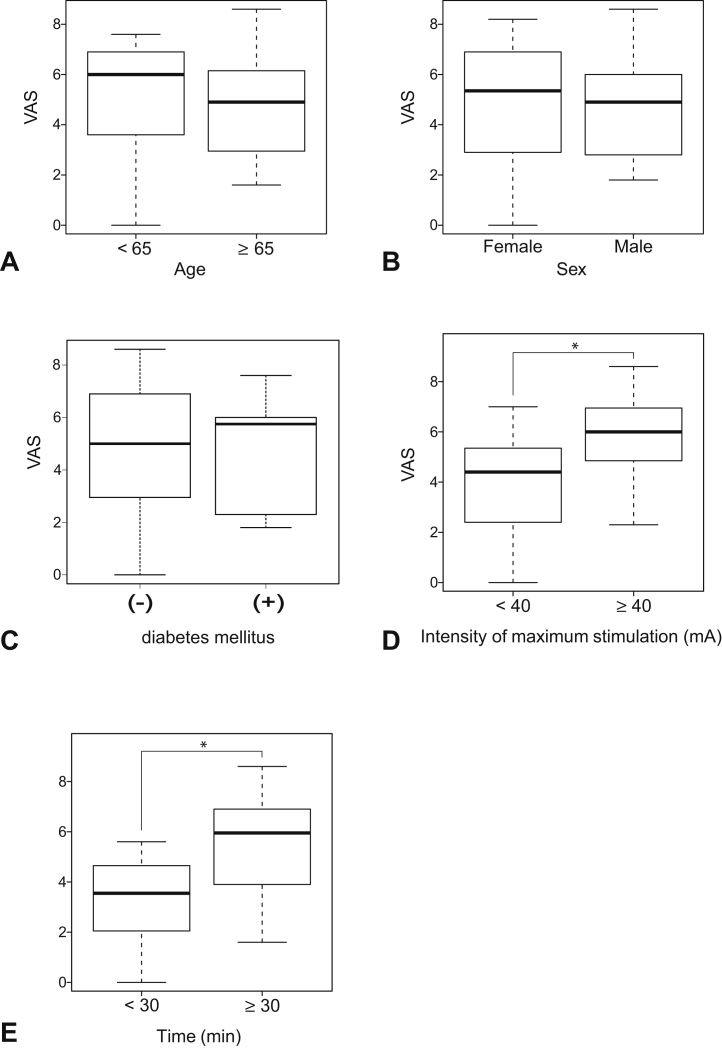
Factors related to NCS-induced pain. **A** No significant difference in pain was identified for age, **B** sex, or **C** diabetes mellitus. The VAS score for pain experienced during NCSs was significantly higher when **D** the intensity of maximum stimulation was ≥40 mA (vs <40 mA; *P* = .025) and **E** when the examination time was ≥30 minutes (vs <30 min; *P* = .012). ∗Statistically significant at *P* < .05, as determined using the Mann-Whitney U test.

The demographic features of patients are presented in Table 1. Analysis of factors related to the VAS score for pain experienced during NCSs showed no significant differences in the VAS score for age (Fig. 1A), sex (Fig. 1B), diabetes mellitus (Fig. 1C), or severity based on the grading scale (*P* = .272) (Table 2). The VAS score for NCSs was significantly higher when the intensity of maximum stimulation was ≥40 mA (vs <40 mA; Fig. 1D) and when the examination time was ≥30 minutes (vs <30 min; Fig. 1E) (Table 2). We performed multiple regression analyses, including VAS as the objective variable, the intensity of maximum stimulation, and the examination time as explanatory variables, and no significant interaction effect was observed from VAS.

**Table 1:** Demographic Characteristics of the Participants∗

	CTS
No. of participants	30
Age, y	69 (60–73)
Sex, female	24
Bland classification	Grade 0: 0
	Grade 1: 1
	Grade 2: 5
	Grade 3: 13
	Grade 4: 0
	Grade 5: 9
	Grade 6: 2
VAS	5.2 (2.9–6.9)
NCS examination time, min	39.5 (28–52)
Intensity of maximum stimulation, mA	37.9 (27.1–44)

∗ Data are presented as the median (interquartile range) or number. Statistical significance was determined by the Mann-Whitney U test.

**Table 2:** Results of Analysis of the Factors Related to NCS-Induced Pain∗

			*P* Value
Age, y	<65	>65	
VAS	NA	4.9 (2.95–6.15)	.59
Sex	Female	Male	
VAS	5.35 (3.0–6.9)	4.9 (3.2–5.8)	.917
Diabetes mellitus	－	＋	
VAS	5.00 (3.02–6.90)	5.57 (3.12–5.97)	.979
Bland classification	Grades 1–3	Grades 4–6	
VAS	4.9 (3.30–5.95)	6.0 (2.95–6.95)	.272
Intensity of maximum stimulation, mA	<40	>40	
VAS	4.4 (2.40–5.35)	6.0 (4.85–6.95)	.025†
Time, min	<30	>30	
VAS	3.55 (2.17–4.52)	5.95 (4.05–6.90)	.012†

NA, not applicable.

∗ Data are presented as median (interquartile range). Statistical significance was determined by the Mann-Whitney U test.

† Significant *P* values.

## Discussion

In this study, we measured the pain because of electrical stimulation experienced by patients with CTS during NCSs. The VAS score was significantly higher for stronger maximum stimulation intensities and longer examination times.

In our results, the mean value of VAS was 5.2. This is similar to the pain of VAS after carpal tunnel release surgery performed under regional venous anesthesia, which has been reported in the past.^13^ Medical staff must recognize that patients feel severe pain during an NCS, which is not considered a treatment procedure.

Several previous studies have attempted to research NCS-related pain. A report of 96 patients with peripheral neuropathy undertook a 6-step survey on pain during NCS/EMG; however, the details of the disease conditions were unclear.^6^ A study including 100 children compared the pain experienced during EMG with that experienced during venipuncture; nevertheless, its accuracy is questionable because of the use of subjective evaluations of children aged 4–17 years.^8^

In a study investigating the association between the VAS scores for pain during NCSs and patient background information (age, sex, height, location, and referral diagnosis) in 108 patients, no obvious factors related to pain were found.^7^ Nevertheless, this past study did not include disease type, stimulation intensity, and examination time. Our study, however, examined age, sex, complications, severity grading scale, maximum stimulation intensity, and examination time concerning the VAS score and showed that the VAS score was higher when the maximum stimulation intensity exceeded 40 mA and when the examination time exceeded 30 minutes. In our hospital, to accurately assess the function of the abductor pollicis brevis muscle, the stimulation is set at a strong enough intensity to ensure that the maximum stimulation intensity is achieved. Although the time required to apply maximum stimulation is short, it is important to recognize that even a short stimulation period can cause subjective pain to the patient and that the subjective pain is more severe with longer examination times. Therefore, clinical technicians must perform the examination smoothly and avoid unnecessarily high intensities to establish an accurate diagnosis. In the multiple regression analysis, no significant difference was observed. Because factors not included in this study may affect the VAS, we need to increase the number of cases and factors in the future.

Our study has several limitations. First, in this study, the VAS after NCSs is compared with the VAS after carpal tunnel release surgery from previous reports. For an accurate comparison, the VAS after NCSs and after carpal tunnel release in the same patient should be compared. Second, in this study, we did not examine the VAS before NCS. For an accurate evaluation, the VAS should have been examined before the test as well. Third, in this study, we did not take the effect of the patients’ ongoing medications into account. The effect of analgesic medication on a patient’s pain ought to be considered.

In conclusion, we assessed pain because of electrical stimulation during NCSs in patients with CTS. The mean value of VAS was 5.2, and the VAS score was higher when the intensity of maximum stimulation was high and when the examination time was prolonged. These findings are important in counseling patients and ensuring that medical staff performs NCS with lower stimulation in a shorter amount of time. Physicians should also inform patients why studies are necessary and beneficial.
